# Well Leg Compartment Syndrome: Pathophysiology, Prevention, and Treatment

**DOI:** 10.3390/jcm11216448

**Published:** 2022-10-31

**Authors:** Matthew Nester, Joseph Borrelli

**Affiliations:** 1 Morsani College of Medicine, University of South Florida, Tampa, FL 33602, USA; 2 Department of Orthopedic and Sports Medicine, Morsani College of Medicine, University of South Florida, Tampa, FL 33602, USA

**Keywords:** well leg compartment syndrome, pathophysiology, oxygen perfusion, compartment pressure, prevention, fasciotomies

## Abstract

The development of compartment syndrome involving the lower limb is a potentially devastating complication of prolonged surgery in patients held in the lithotomy position. Well leg compartment syndrome (WLCS) was recognized in 1953. The incidence of this condition has been reported to range from 0.20% to 0.03%. The mechanism of WLCS development in the absence of trauma appears to be related to prolonged hypoperfusion of the limb, pressure on the muscle compartments, and in some cases, reperfusion of the ischemic limb. This grave complication develops either during or immediately after prolonged surgery in which the patient was held in the Lloyd-Davies lithotomy or hemi-lithotomy position. Surgeons must be aware of the potential for WLCS development during prolonged surgery. Signs of developing WLCS include swelling, increased firmness of the muscle compartments, discoloration, and cooling of the limb. Preventive measures can be taken without contaminating the surgical field by returning the limb to the right atrium level. Once the diagnosis has been made, failure to prevent the development of WLCS requires extensile fasciotomy of each leg compartment to restore perfusion and relieve elevated intra-compartment pressures. This article reviews the pathophysiology, prevention, and treatment of WLCS.

## 1. Introduction

Acute compartment syndrome (ACS) occurs when there is an increase in tissue pressure (commonly referred to as intra-compartment pressure) within a fascial compartment sufficient to compromise local circulation. When the intra-compartment pressure exceeds the perfusion pressure within the compartment, the neuromuscular function is compromised, and if allowed to continue, the viability of the compartment’s muscles and nerves is threatened [1,2,3,4,5]. Circulatory patency is important for maintaining normal tissue function, including that of nerves and muscles. ACS is most associated with blunt-force trauma. This trauma results in swelling, direct tissue damage, inflammation, and local ischemia and leads to patchy oxygen metabolism deficiencies, leading to increased intra-compartment pressures [5]. Several investigators have attempted to measure normal intra-compartment pressures with a variety of different techniques. Currently most clinicians feel as if the normal interstitial fluid pressure within relaxed compartments is approximately <20 mmHg in adults [6]. As the intra-compartment pressure approaches and subsequently exceeds normal tissue perfusion pressures, blood flow within the compartment is slowed and may ultimately be stopped. As the perfusion pressure within the compartment approaches and exceeds the intra-compartment pressure, ischemia of the compartment contents develops. If ischemia persists for a prolonged period, loss of nerve and muscle function and viability will follow the development of ACS.

The devastating effects of an undiagnosed or “neglected” compartment syndrome have been recognized for quite some time. The earliest report was published by Richard von Volkmann in 1881 [7]. In that case, von Volkmann described the presence of myonecrosis and contracture of the arm due to prolonged muscle ischemia. Since this early report physicians have come to realize that not only are ACS associated with myonecrosis and contractures left untreated rhabdomyolysis, myoglobinuria and renal failure may also develop threatening the life of the patient. Moreover, no one is immune to this potentially devastating condition. Compartment syndromes are known to occur in all age groups, from infants to the very old, according to gender, race, and ethnicity. Young active individuals who sustain high-energy trauma are most likely to develop ACS.

ACS has been identified in various scenarios. In each situation, the common pathway is compromised blood flow (perfusion pressure) within the osseous fascial compartment(s). ACS is typically associated with traumatic injury (with or without fracture). However, it is also known to develop because of significant swelling or bleeding into the fascial compartment(s) or external pressure (from circumferential burns, casts, and wraps) of the injured limb. Additionally, ACS is known to occur because of the formation of a space-occupying mass (for example, infection and wayward infusion) within a fascial compartment and follow acute limb ischemia with reperfusion injury. Exertional compartment syndrome has also been reported. This type of ACS results from the swelling of the muscles within the osseous-fascial compartment due to strenuous exercise.

Well leg compartment syndrome (WLCS) has also been identified in various clinical settings. WLCS develops in a previously uninjured extremity (most often the leg), most commonly because of prolonged static positioning of a patient’s limbs on an operating room table. This positioning is believed to diminish the perfusion pressure within the fascial compartments of the uninjured limb, causing the development of increased intra-compartmental pressures and, in some cases, WLCS. Once an ACS has developed, whether it be an exertional CS or WLCS, it is critical to remember that “time is tissue” and that the sooner the compartment pressures are normalized, and normal perfusion is re-established within the compartments, the better the chance of limiting tissue damage, and the better chance of preserving limb function. This article reviews the pathophysiology, prevention, and treatment of WLCS.

## 2. Well Leg Compartment Syndrome

Gordon et al., in 1953, were credited with the first description of WLCS (at that time, it was referred to as lower nephron syndrome) following spinal surgery in which the patient was positioned prone with their knees and hips maximally flexed. Since then, WLCS has been reported as a complication associated with general surgery (both open and laproscopic surgery), colorectal surgery, urological surgery (including robot-assisted radical cystectomy), obstetric and gynecological surgery, orthognathic surgery and orthopedic surgery [8,9,10,11,12,13]. Although ischemia of the muscles and nerves within the affected compartments is the ultimate culprit, unlike the most common causes of ACS (trauma, crush injury, and fracture), WLCS develops in an uninjured limb. Therefore, the origin of WLCS differs considerably from that of the more commonly encountered ACSs, although the final common pathway is the same (hypoperfusion leading to muscle and nerve damage).

**Figure 1:**
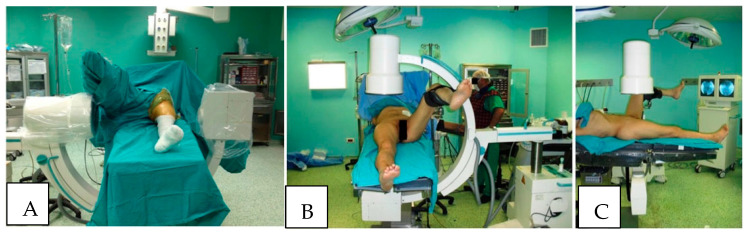
(**A**) Typical patient positioning in the hemi-lithotomy position for intramedullary nailing and open reduction and internal fixation of proximal femur fractures. The well-leg (L) is held flexed at the hip and knee in a generic “well-leg” holder. The operative leg is positioned with the hip and knee extended. The C-arm can be positioned (**B**,**C**) to allow an unobstructed image of the hip in both the AP and cross-table lateral images.

Over the past several decades, factors contributing to the development of WLCS have been investigated. Although WLCS has been reported in at least two patients who underwent surgery in the lateral decubitus position, most reported cases of WLCS have been in patients who were positioned in the lithotomy or hemi-lithotomy position during surgery [8,9,10,11,12,13,14,15]. In the hemi-lithotomy position, commonly used in orthopedic surgery, particularly during antegrade femoral nailing and surgery of the hip and proximal femur fracture, the “well leg” is flexed at the hip and knee, and the hip is abducted and externally rotated. The leg is generally held in this position with a platform positioned beneath the “well leg” calf. This position is favored by many orthopedic surgeons to facilitate the use of fluoroscopic image intensifiers during the operative stabilization of the proximal femur and hip (Figure 1). This position has been shown to result in decreased perfusion pressure, oxygenation of the leg compartments, and increased intercompartmental tissue pressures. If these conditions are allowed to persist for a prolonged period, decreased perfusion and oxygenation, as well as increased compartment pressures, have been reported to lead to the development of WLCS [16,17,18,19,20,21].

## 3. Normal Intra-Compartmental Pressures

Prayson et al. established a baseline for intra-compartmental pressures and used the uninjured limb of patients being treated for an isolated (contralateral) lower extremity fracture. In that investigation, the average compartment measurements were 16.6 ± 7.5 mmHg (range 3–40 mmHg) [3,22].

## 4. Pathophysiology and Influencers of WLCS

There have been several investigations into the changes that occur in perfusion, oxygenation, and intra-compartmental tissue pressures when the leg is held at various heights relative to the level of the heart. Based on one of such investigations, the perfusion pressure within the leg was shown to decrease by 0.78 mmHg for each centimeter that the leg is elevated above the right atrium of the heart. In that study, it was determined that perfusion in each compartment was reduced by approximately 24 mmHg when the leg was elevated above the right atrium by 30.5 cm (1 ft) [23]. It has also been shown that performing surgery under relative hypotension, whether intentionally or related to the patient’s injuries, further reduces the perfusion pressure of the leg [24]. These findings should be considered when operating on a patient in the lithotomy or hemi-lithotomy positions.

**Figure 2:**
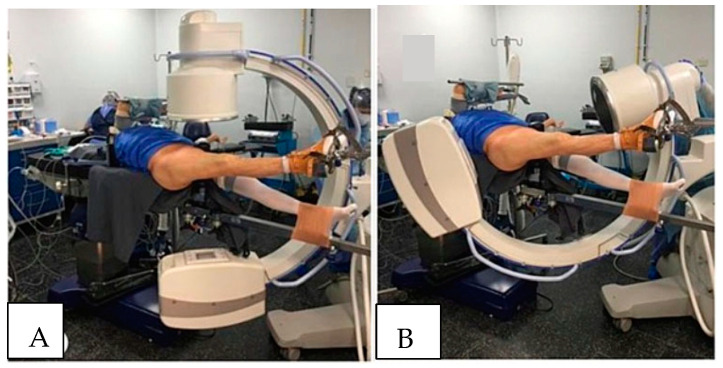
Typical patient positioning in the hemi-lithotomy position for intramedullary nailing and open reduction and internal fixation of proximal femur fractures. (**A**) The well-leg (L) is held flexed at the hip and knee in a generic “well-leg” holder. The operative leg is positioned with the hip and knee extended. (**B**) The C-arm can be positioned to allow an unobstructed image of the hip in both the AP and cross-table lateral images.

WLCS is best avoided by limiting the need to position the non-operative leg above the right atrium if possible (Figure 2A,B). If the leg, or legs, must be positioned above the level of the right atrium, then limiting the time the leg is elevated should be kept to no more than 3 h in normotensive patients. It is imperative that surgeons and procedurists who routinely treat patients in the lithotomy or hemi-lithotomy position be aware of the possibility that WLCS can develop. By remaining cognizant of this possibility, clinicians can take appropriate steps to prevent the development of WLCS. Ideally, the lithotomy and hemi-lithotomy positions should be avoided, particularly if the planned procedure is likely to take more than 3 to 4 h to complete. In certain operative procedures, including surgery on the contralateral hip and proximal femur, rather than placing the “well leg” in the lithotomy position, the legs can be “scissored.” To “scissor” the legs, the “well leg” is extended at the hip and knee, and the hip extended to position so that the “well leg” is, for the most part, below the level of the operative leg. Scissoring the legs, as opposed to flexing the hip and knee of the “well leg” and placing the leg on a “well leg” holder, should prevent the development of WLCS, as perfusion pressures are generally not affected. If the lithotomy or hemi-lithotomy position is necessary, hip flexion should be kept to less than 90°, and the platform supporting the calf should be positioned so that it does not apply pressure on the popliteal fossa. Different types of “well leg” holders are commercially available, and certain holders have been shown to increase intra-compartment tissue pressure more than others. These holders have been shown to lessen intra-compartment tissue pressures in the leg and decrease the risk of developing WLCSs.

The choice of “well leg” holder should be considered when preparing for surgery with the patient in the lithotomy or hemi-lithotomy position. Pfeffer et al. found that the patient being in the lithotomy or hemi-lithotomy position with the “well leg” supported by the calf and knee increased intra-compartment pressure to 16.5 ± 3.4 versus 10.7 ± 5.8 mmHg when the leg was not elevated (mean ± SD; *p* < 0.05). The addition of intermittent compression decreased pressures to 13.4 ± 5.1 mmHg while in the lithotomy position (*p* < 0.05) and to 9.1 ± 7.0 mmHg when the leg was not elevated (*p* < 0.05). In contrast, the lithotomy position with support near the ankle decreased intra-compartment pressure to 8.7 ± 5.6 versus 13.3 ± 5.1 mmHg in the supine position (*p* < 0.05). The addition of intermittent compression devices decreased pressure further to 6.5 ± 5.4 mmHg in the lithotomy position (*p* < 0.05) and to 10.3 ± 4.7 mmHg in the supine position (*p* < 0.05). These investigators concluded that the lithotomy position is associated with increases in intra-compartment pressures and that the degree is dependent on the method of leg support used [20]. Furthermore, they indicated that intermittent external compression (sequential compression devices) further reduced intra-compartment pressures in the lower leg. Therefore, increases in intra-compartment pressure during surgery in the lithotomy position with the calf or knee support contribute to the increase in the intra-compartmental pressures and may contribute to the development of WLCS. Notably, the use of intermittent external compression of the leg may help reduce the increase in intra-compartmental pressures and may help avoid the development of WLCS.

## 5. Preventing Development of WLCS

The idiom: “an ounce of prevention is worth a pound of cure” (Benjamin Franklin famously advised Philadelphians regarding preventing fires in 1736) is applicable in settings where the development of WLCS is possible. Preventing the development of an uncommon but potentially limb and even life-threatening complication should be a priority.

Although no fail-safe protocol has been developed to prevent the development of a WLCS during surgery, Addley et al., have proposed a peri-operative check-list and risk-reduction protocol based upon known risk factors. Based upon their extensive experience with treating patients in the lithotomy position during gynecological surgery they’ve identified several risk factors, including: age < 35, diabetes mellitus, hypothyroidim, bleeding diatheses, pre-existing vascular disease and intra-pelvic pathology, increased muscle mass or deformity of the lower extremity and BMI greater than 25 kg per meter^2^.

Although their “Peri-operative risk-reduction and ‘WELL_LEG’ check list has not been evlauted for its effectiveness in preventing the development of a WLCS during surgery they suggest. (1) identification of patient’s risk factors prior to surgery, (2) pre-operative team briefing, (3) pro-active approach to homeostasis by the anesthesia team to maintain normal blood pressure during the procedure, (4) thrombo = prophylaxis measures be taken with intermittent compression devices, (5) patient be positioned on an anti-slip gel mat by the senior member of the operative team, (6) intra-operative ‘leg-checks’ and ‘leg-rests’ performed at 2 h intervals by an unscrubbed member of the operative team, (7) post-operative assessment of the limb for the symptoms and signs of a WLCS at 2 h intervals for 4 h [25].

**Figure 3:**
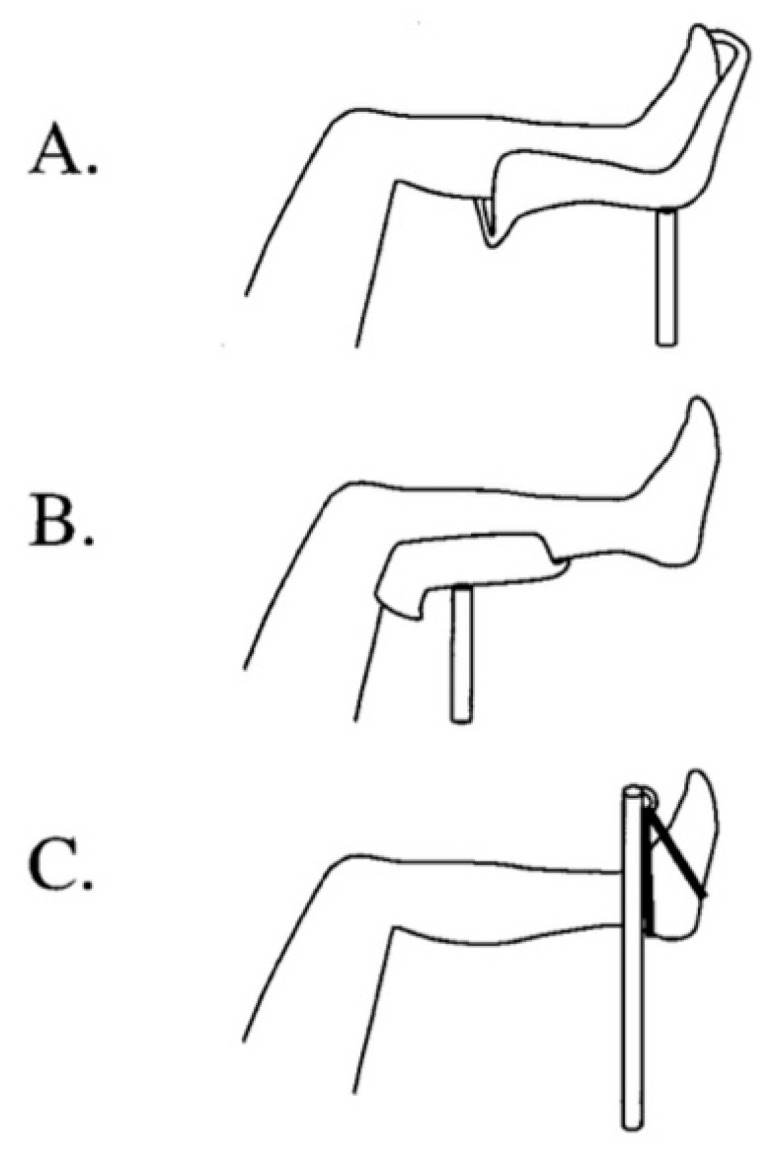
Three different types of well-leg holders. (**A**) the Allen stirrup system, with support of the limb in a boot-like device, (**B**) generic knee support which supports the distal thigh, knee and leg, (**C**) a cloth sling around the ankle and foot attached to a padded vertical “candy cane”.

Others have also worked on algorithms for preventing the development of WLCS has not yet been established, work in this area is promising. Hara et al. studied the effects of the following: 1. Changing from the lithotomy position to the “open-leg position”; 2. Relieving excessive pressure on the leg contact area when the patient is in the lithotomy position (Figure 3). 3. Limiting leg elevation relative to the height of the patient’s right atrium; 4. Maintaining normal blood pressure during surgery; 5. Horizontally repositioning the operating table every 3 h to maintain blood flow to the leg; and 6. Decompressing the contact area of the leg when the patient is in the lithotomy position during surgery [26].

To prevent the development of WLCS, the surgeon and the entire operative team must be aware of its potential. Therefore, the surgeon and team must first know that WLCS can develop and must remain vigilant of this possibility. Intraoperatively, the “well leg” positioned in the lithotomy position is generally maintained under sterile drapes and out of sight of the surgeon, anesthesiologist, and the rest of the operating room personnel. However, this does not exonerate the operating team from monitoring the “well leg” and addressing its concerns. As in almost all processes of developing compartment syndromes, there are generally changes that can be observed in the leg before the onset of myonecrosis. Moreover, since the surgical patient is incapacitated, symptoms of developing WLCS cannot be obtained directly from patient complaints. Therefore, the leg must be monitored closely for the development of signs of hypoperfusion and possibly the development of WLCS. These signs include swelling of the leg, increased firmness of the leg compartments (as determined by direct palpation of each of the muscle compartments), changes in the coloration of the limb (“dusky” or pale appearance) and lowering of the temperature of the skin. Individually, each of these signs may indicate hypoperfusion of the leg. As the number of these findings increases, there is an increasing likelihood of hypoperfusion of the limb, and steps should be taken to reverse these signs to decrease the likelihood of developing WLCS. Intra-compartmental pressure measurements can be taken to objectively assess compartment pressures and, when assessed in combination with physical findings, go a long way in the diagnosis of WLCS.

Intraoperative maneuvers to identify the possibility that WLCS is developing should begin to be performed at least at the 3 h time point and subsequently. Moreover, these maneuvers must be coordinated with the operative team while maintaining the sterility of the operative field. These maneuvers include visualization of the leg and palpation of the limb to assess the development of swelling and firmness of the compartments. Palpation should also include assessing the pulses of the foot, although WLCS can develop in the setting of palpable pulses, and one should not wait until the pulses are not palpable before recognizing that WLCS has developed.

If WLCS is suspected of developing intraoperatively or shortly after while the patient is still anesthetized or in the recovering phase, intra-compartmental pressure measurements should be performed. Intra-compartmental pressure measurements are often used to confirm clinical suspicion, particularly in obtunded patients.

## 6. Diagnosing WLCS

Unlike ACS, which develops due to an unplanned traumatic injury or a complication of medical treatment, the possibility of the development of WLCS can be anticipated. As outlined above and in previous reports in the literature, there are identifiable risk factors for the development of WLCS. Most noticeably, the positioning of a patient in the lithotomy or hemi-lithotomy position for greater than 3–4 h. Maintaining hip and knee flexion at ≥90° for 3–4 h is a known risk factor for the development of WLCS. The use of a “well leg” holder that applies pressure along the length of the “well leg” calf and pressure in the popliteal space is also a risk factor and has been shown to decrease perfusion pressure within calf compartments. Relative intraoperative hypotension may also contribute to the development of WLCS, particularly in the setting of prolonged hip and knee flexion (lithotomy position) and the use of a conforming leg holder.

## 7. Measurement and Interpretation of Intra-Compartmental Pressures

In the past, an absolute pressure value was used to confirm or refute the presence of compartment syndrome. A measurement of ≥30 mmHg was previously used to confirm the diagnosis. Unfortunately, using an absolute threshold value was found to result in a relatively high rate of false positives and led to unnecessary fasciotomy. Intra-compartment pressure (ICP) has also been directly compared with diastolic pressure. When the measured ICP was found to be within 10 to 30 mmHg of the patient’s diastolic blood pressure, clinicians hypothesized that CS existed [27]. Recently, clinicians have begun to use delta pressure to confirm or refute the presence of CS. Delta pressure = diastolic pressure—the measured intra-compartmental pressure. A delta pressure of ≤30 mmHg is often used to confirm the presence of a CS and the need for fasciotomy [28,29].

A commercially available stick catheter (Stryker Surgical, Kalamazoo, MI, USA) is commonly used to augment clinical findings and to facilitate the direct measurement of intra-compartment pressures. These measurements are particularly useful in the obtunded, intubated, unconscious or uncooperative patient. This device allows a quick and simple means to accurately measure intra-compartmental pressures. Once assembled, according to the manufacturer’s instructions, the skin overlying the areas are cleansed with an antiseptic cleanser. The needle of the assembled and calibrated pressure monitor is held perpendicular to the compartment and inserted as gently as possible through the skin to a depth appropriate for the target compartment. A 0.3 mL volume of saline is slowly injected into the compartment. Once the recording displayed in the window of the device has equilibrated, the pressure (mmHg) of each compartment is recorded. All four compartments of the leg and if clinically indicated each compartment of the thigh should also be measured. If necessary, normal compartments of the opposite leg or upper extremities can be used as a control to assure the device is functioning normally.

Indwelling pressure monitoring catheters have also been developed to measure intra-compartmental pressures in real-time. These devices allow minute to minute monitoring of intra-compartmental pressures and when compared with changes in a patient’s systemic blood pressure can facilitate the diagnosis of an ACS at its onset [30,31,32].

Newer means of determining the presence of an ACS are being investigated. These “higher tech” devices are designed to be less invasive and improve our abilities to diagnosis an ACS early before irreversible muscle and nerve damage has occurred. These newer methods, which have not been validated for clinical use at this time, include near-infrared spectrometry (NIRM), measurement of intramuscular glucose and oxygen tension levels, ultrasound measurements and monitors to detect changes in muscle microvascular blood flow, oxygenation, pH and perfusion pressure [33,34,35,36].

## 8. Treatment for WLCS

When it is determined that WLCS has developed, a 4-compartment fasciotomy of the leg should be performed immediately. There are four well-defined compartments within the leg. The anterior compartment, which is situated just lateral to the tibial crest and anterior to the fibula, contains the extensor hallicus longus, extensor digitorum communis, tibialis anterior, and peroneus tertius. The deep peroneal nerve and the anterior tibial artery also lie within this compartment. The lateral compartment contains the peroneus brevis and longus and is localized along the lateral aspect of the leg in a compartment defined anteriorly by the anterior intermuscular septum and posteriorly by the posterior intermuscular septum. The lateral compartment also contains the superficial peroneal nerve and a portion of the deep peroneal nerve before passing into the anterior compartment. The posterior compartment is organized into superficial and deep posterior compartments. The superficial compartment contains the gastrocnemius, soleus, plantaris, and sural nerves. The deep posterior compartment contains the tibialis posterior, flexor hallucis longus, flexor digitorum longus, popliteus, tibial, and posterior tibial arteries. The peroneal artery, which is a branch of the popliteal artery, also resides within the deep posterior compartment.

Although different approaches for complete decompression of each compartment have been described, many authors prefer a two-incision technique to ensure thorough decompression of each compartment. To decompress the anterior and lateral compartments of the leg, a 15–20 cm incision can be made longitudinally along the lateral aspect of the leg parallel to the intermuscular septum. Care must be taken to avoid injury to the peroneal nerve proximal and distal to the superficial peroneal nerve. Through this incision, the anterior and lateral compartments would be completely decompressed. Once decompressed, each muscle belly should be assessed for color, contractility, and bleeding, and these findings should be documented accordingly.

**Figure 4:**
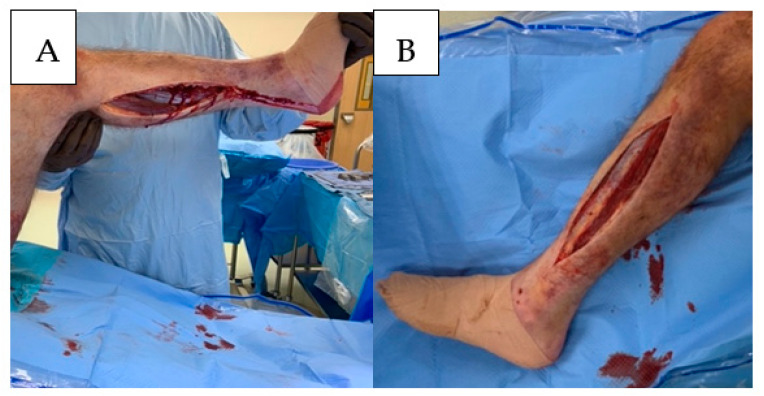
Four compartment fasciotomies performed with a medial (**A**) and lateral (**B**) fasciotomy incisions.

The superficial and deep posterior compartments can be decompressed via a single incision positioned 1–1½ finger breaths posterior to the medial edge of the tibial shaft. Care should be taken to avoid injury to the saphenous veins and nerves. Decompression of the deep compartment can be performed by elevating the soleus attachment to the posterior tibia. Release of the posterior-superficial compartment is accomplished by incising the enveloping fascia of the gastrocnemius muscle, the length of the muscle belly, and the musculotendinous junction. The viability of these muscle bellies should be assessed, and the findings should be documented (Figure 4).

## 9. Conclusions

Fortunately, WLCS rarely develops. However, because of the potentially devastating consequences of WLCS, surgeons and their surgical teams must remain vigilant in its development. Based on the available literature, WLCS most commonly develops in patients maintained in the lithotomy position during surgery (including orthopedic, general, urological, and gynecologic surgeries). The full lithotomy position is thought to lead to WLCS by causing an increase in muscle pressure in the lower leg, a decrease in the arterial perfusion pressure of the limb, and a decrease in SpO_2_ within the leg because of its position above the right atrium and associated kinking of the blood vessels [16,17,18,19,20,21,22,23,24]. WLCS may also develop postoperatively in patients in the PACU owing to reperfusion after prolonged hypoperfusion while in the lithotomy position. Clinical symptoms of WLCS may include leg pain (in conscious patients), leg swelling, increased firmness of the leg compartments, and changes in the appearance and temperature of the legs. The presence of WLCS, when suspected clinically, can be confirmed by the direct measurement of the intra-compartmental pressures within each of the compartments in the leg, as well as when indicated in the thigh.

Understanding how and why WLCS develops can inform surgical teams about its prevention. Hara et al. investigated how to prevent the development of WLCS in a cohort of 1,951 patients undergoing lengthy operative procedures while in the lithotomy position [28]. They found that avoiding the lithotomy position effectively decreased the risk of developing WLCS. In addition, if the patient must be positioned in the lithotomy position, then the “well leg” should be monitored closely, and the leg should be taken down from the lithotomy position for at least 20 min at regular intervals beginning at the 3 h mark, to restore normal perfusion pressures in the leg. These investigators also demonstrated that relieving or avoiding pressure on the “well leg,” limiting leg elevation by limiting hip and knee flexion, maintaining normal blood pressure during surgery, and repositioning the operating table periodically during the procedure also decreases the likelihood of developing WLCS.

Intraoperative leg monitoring is essential for early recognition of the development of WLCS. Vigilance must also be continued after the patient has returned to the supine position and brought to the post-anesthesia recovery room, as WLCS can also fully develop upon reperfusion of the leg. When WLCS is diagnosed, emergent treatment with 4-compartment fasciotomies must be performed. These fasciotomies must completely decrease intra-compartmental pressures in the leg and re-establish normal perfusion pressures.
